# Thermal Inactivation Kinetics and Radio Frequency Control of *Aspergillus* in Almond Kernels

**DOI:** 10.3390/foods11111603

**Published:** 2022-05-29

**Authors:** Yu Gao, Xiangyu Guan, Ailin Wan, Yuan Cui, Xiaoxi Kou, Rui Li, Shaojin Wang

**Affiliations:** 1 College of Mechanical and Electronic Engineering, Northwest A&F University, Xianyang 712100, China; gaoyu2001@nwafu.edu.cn (Y.G.); xiangyuguan@nwafu.edu.cn (X.G.); kouxiaoxi@nwafu.edu.cn (X.K.); 2 College of Food Science and Engineering, Northwest A&F University, Xianyang 712100, China; wanailin221@nwafu.edu.cn (A.W.); cuiyuan0507@nwafu.edu.cn (Y.C.); 3 Department of Biological Systems Engineering, Washington State University, Pullman, WA 99164-6120, USA

**Keywords:** almond kernels, *Aspergillus*, radio frequency, thermal inactivation kinetics, verification

## Abstract

Mold infections in almonds are a safety issue during post-harvest, storage and consumption, leading to health problems for consumers and causing economic losses. The aim of this study was to isolate mold from infected almond kernels and identify it by whole genome sequence (WGS). Then, the more heat resistant mold was selected and the thermal inactivation kinetics of this mold influenced by temperature and water activity (*a_w_*) was developed. Hot air-assisted radio frequency (RF) heating was used to validate pasteurization efficacy based on the thermal inactivation kinetics of this target mold. The results showed that the two types of molds were *Penicillium* and *Aspergillus* identified by WGS. The selected *Aspergillus* had higher heat resistance than the *Penicillium* in the almond kernels. Inactivation data for the target *Aspergillus* fitted the Weibull model better than the first-order kinetic model. The population changes of the target *Aspergillus* under the given conditions could be predicted from Mafart’s modified Bigelow model. The RF treatment was effectively used for inactivating *Aspergillus* in almond kernels based on Mafart’s modified Bigelow model and the cumulative lethal time model.

## 1. Introduction

Almonds are rich in unsaturated fatty acids, a variety of vitamins and trace elements, and are accepted and loved by consumers around the world. Global almond production in 2020 was approximately 4.14 million metric tons reported by the Food and Agriculture Organization (FAO, Rome, Italy), and the United States, Spain, Australia, Iran and Turkey are the top five product-consuming countries [1]. However, potential mold contamination in almonds is considered a very serious food safety problem all around the world. Molds in low moisture foods can survive for quite a long period and may grow quickly once the storage environment becomes appropriate, thereby causing great quality degradations and economic losses. Therefore, it is of great significance and urgency to eliminate molds in almonds and almond products during storage, production and processing.

RF heating has already been applied to control the population of insect pests and pathogens in a wide variety of agricultural products owing to its characteristics of volumetric heating, deep penetration, short treatment time, no chemical residues, and no noteworthy quality loss [2,3,4,5,6,7]. Proper RF treatment parameters (heating temperature and time) can effectively avoid safety problems caused by insufficient heating and food quality deterioration made by excessive heating [8]. Since different molds have different heat resistance under different environmental factors and food compositions [9,10,11], the detailed information on almond molds and their heat resistances influenced by temperature and *a_w_* is limited. Therefore, it is essential to identify the almond mold species and evaluate the thermal inactivation kinetics of molds influenced by temperature and *a_w_* before RF validation [12].

The thermal inactivation kinetics of mold is usually determined under isothermal conditions. The test cells developed by our laboratory [13] may provide nearly isothermal conditions with fast heating or cooling rates and good heating uniformity and could be potentially used for acquiring thermal inactivation kinetics of target mold inoculated in almond kernel flour. The first-order kinetic, Weibull [14,15,16], and Mafart’s modified Bigelow models [17,18] were applied to describe the thermal inactivation kinetics of molds after thermal treatments under isothermal conditions. For real practical thermal treatments with non-isothermal performances, the inactivation rate of the target microorganism was evaluated by the cumulative thermal lethal time model [8,19,20].

In the actual process of RF pasteurization, the effects of the non-isothermal treatment stage during heating up and the isothermal treatment stage during holding should be comprehensively considered regarding mold inactivation. The cumulative thermal lethal model is useful to guide the development of the RF treatment protocol and further determine the total RF process time for achieving the required inactivation level of mold in almonds.

The objectives of this study were: (1) to isolate mold from infected almond kernels in cold storage conditions and identify mold species using the whole genome sequence (WGS); (2) to compare the heat resistance of the isolated molds and develop the thermal inactivation kinetic models of the selected more thermal-resistant mold (*Aspergillus*) as influenced by three temperatures and three *a_w_* levels; and (3) to verify the inactivation rate of the target mold in almonds when subjected to hot air-assisted RF treatments using the developed thermal inactivation kinetic model.

## 2. Materials and Methods

### 2.1. Sample Preparation

About 30 kg raw and dried almond kernels (Nonpareil) were bought from Paramount Farming Company (Modesto, CA, USA). The incomplete and damaged almond kernels were eliminated, then the polyethylene bags were used for sealing intact almond kernels, and the refrigerator (BD/BC-297KMQ, Media Refrigeration Division, Hefei, China) at 4 ± 1 °C was used for storing these almond kernels. The almond kernels’ initial moisture content (MC) was 3.91 ± 0.12% on wet basis (w.b.), which was determined by a moisture analyzer (HE53, Mettler-Toledo, Shanghai, China). The MC of almond kernels was adjusted to three different levels of MC or *a_w_* by directly adding pre-calculated distilled water for studying the effect of MC or *a_w_* levels on molds’ thermal inactivation efficacy. The adjusted almond kernels were sealed into polyethylene bags for at least 5 d at 4 °C and shaken at least 3 times each day to obtain the almond kernels with a sufficiently even MC distribution. After the almond kernels were adjusted to the predetermined MC, the kernels were grounded with a grinder until their flour could pass through No.18 sieve (Aperture size was 1 mm, corresponding to 16 Taylor sieve). The water activity meter (Aqua Lab 4 TE, Decagon Devices, Inc., Pullman, WA, USA) was used for measuring the *a_w_* of almond kernels.

#### 2.1.1. Isolation of Spoilage Molds

About 200 g almond kernels were randomly selected from samples stored in the refrigerator and then their MC was adjusted to 10.11% (w.b.) and stored in a 25 °C incubator (LRH-250, Zhujiang, Guangdong, China) for 15 d, moldy almond kernels appeared. About 25 g moldy almond kernels were immersed into 95% ethyl alcohol for sterilization, and then put into 225 mL normal saline and shaken fully for about 30 min [13]. Next, the suspension was transferred into Potato Dextrose Agar (PDA; Beijing Land Bridge Technology Co., Ltd., Beijing, China) and Czapek Yeast Extract Agar (CYA; Beijing Land Bridge Technology Co., Ltd., Beijing, China) media, respectively. All the media were monitored for about 5 d in a biochemical incubator (LTH-100, Shanghai Longyue Instrument Equipment Co., Ltd., Shanghai, China) at 29 ± 0.5 °C. Two single pure isolated colony types were obtained by conducting gradient dilution and streaking plate method on mixed colonies and then identified by WGS.

#### 2.1.2. Preparation of Mold Suspension

The molds of *Penicillium* and *Aspergillus* were identified by WGS. The strains of *Penicillium* and *Aspergillus* were cultivated on CYA and PDA media, respectively. The two strains on different media were incubated at 29 ± 0.5 °C for 5 d in the biochemical incubator. Conidia were gently scraped off from the surfaces of the 5-day-old cultures using a spreader after pouring sterile 0.85% isotonic NaCl solution on the cultivated agar. The conidia’s population was adjusted to 1 × 10^10^ CFU/mL in both suspensions for further use.

### 2.2. Thermal Treatment

**Figure 1:**
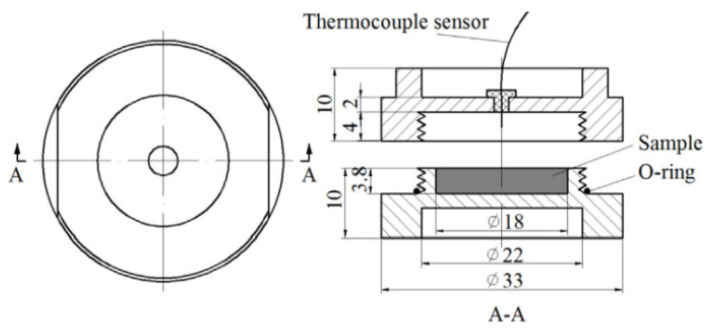
Schematic view of a test cell with 18 mm diameter and 3.8 mm height (All dimensions are in mm) (Adapted from Hou et al. [13]).

Custom-designed test cells were used for conducting the isothermal treatment (Figure 1), which were successfully used for studying the thermal inactivation kinetics of *Penicillium* in chestnuts [13]. These test cells’ detailed information can be found in Hou et al. [21]. Before inoculation, the test cells and almond kernel flour were sterilized at 121 °C and 105 °C for 20 min and 10 min by a vertical autoclave (LMQ.C, Shinva Medical Instrument Co., Ltd., Shandong, China), respectively. Then, 0.88 ± 0.03 g almond kernel flour was put into test cells and 20 μL mold suspension was inoculated into almond kernel flour. Then the test cells were left inside a biosafety hood at 25 °C for 1 h to achieve moisture equilibrium before hot water treatments. After that, the test cells were immersed and heated in a preheated water bath (YT-10A, Beijing Yatai Cologne Experimental Technology Development Center, Beijing, China). The treatment time started from the moment when the central temperature of the suspension reached the set temperature value, and the temperature fluctuation did not exceed ±0.5 °C, which could be considered near-ideal isothermal conditions. The sample temperature was monitored from one cell filled with uninoculated almond kernel flour by type-T thermocouples (HH-25TC, Omega Engineering Ltd., Stamford, CT, USA).

Based on the preliminary results, 62 °C + 5 min, 65 °C + 3 min, and 68 °C + 1 min were selected for comparing the heat resistance of two molds isolated from moldy almond kernels. Then, the higher heat resistance mold in almond kernels was chosen to obtain the thermal inactivation kinetics for further RF pasteurization validation. Three *a_w_* levels of 0.657, 0.854, and 0.923 corresponded to sample MC of 5.82%, 10.11%, and 13.85% w.b. were used to determine the *a_w_* effect on inactivation of the target molds at three target temperatures. To achieve at least 4 log reductions of the target mold for thermal inactivation kinetic determination and further for RF pasteurization validation, 59, 62 and 65 °C for *a_w_* of 0.923, 62, 65 and 68 °C for *a_w_* of 0.854, or 65, 68 and 71 °C for *a_w_* of 0.657 were selected. For comparing the *a_w_* influence on the target mold inactivation, 65 °C was included at each *a_w_*.

After holding different time intervals, the test cells with inoculated almond kernel flour were placed into cold water (≤4 °C) over 3 min before further analysis. One test cell with inoculated almond kernel flour without thermal treatment served as control. The total population of colonies in the control and heat-treated samples was counted and compared for evaluating thermal treatment effects.

Almond kernel flour was scraped into sterile 0.85% NaCl solution and shaken for at least 3 min. A total of 100 μL of the solution was then added to 0.9 mL sterile NaCl solution for 10-fold serial dilutions until suitable countable numbers were reached. Finally, 100 μL of each dilution was evenly spread on the cultivated agar and 29 °C incubation for about 2 d. Colony counts were obtained by plate counting.

### 2.3. Thermal Inactivation Kinetics Model

The thermal inactivation kinetics was described by the first-order kinetic and the Weibull distribution. The equation of the first-order kinetic model was presented below [17]:

(1) $$\log\;\frac{N}{N_{0}}=-\frac{t}{D}$$

where *N* and *N*_0_ are the mold populations (CFU/g) at time *t* and initial time, *t* means isothermal treatment holding time (min), and *D* is a decimal time (min) for 1 log reduction of the microbial population at a required temperature (°C).

The equation of the Weibull distribution model was described as follows [22,23,24]:

(2) $$\log\;\frac{N}{N_{0}}=-\;(\frac{t}{\delta }{)}^{p}$$

where *δ*-value is a scale parameter that primarily represents the survival curve steepness. The *p*-value is a shaped parameter, and may be linear (*p* = 1) or nonlinear (*p* < 1 or *p* > 1). The suitability of the models can be evaluated by the coefficient of determination (R^2^) and root mean square error (RMSE).

### 2.4. Effects of Temperature and a_w_ on Thermal Inactivation Kinetic Model

The secondary model was usually applied to characterize the influence of temperature (*T*) or *a_w_* of the samples on the parameters of kinetic model [25,26]. The model of simplified Mafart’s modified Bigelow in references [8,17] was depicted as follows:

(3) $$\log\;\frac{D}{D_{\mathrm{ref}}}=-\frac{{(T\;-\;T}_{\mathrm{ref}})}{z_{T}}-\frac{\left(a_{w}-a_{\mathrm{wref}}\right)}{z_{a_{w}}}$$

where *D_ref_* is the decimal time (min) reducing 1 log population at *T_ref_* (65 °C) and *a_wref_* (1.00), *z_T_* and *z_aw_* are temperature (°C) and *a_w_* increments, respectively, required to reach 90% *D*-value reduction of target microorganisms.

### 2.5. Determining Cumulative Time–Temperature Effects

The lethal effect of heating up and isothermal time can be explored from the cumulative lethal time model during the whole thermal treatment. At a reference temperature *T_ref_* (°C), the equivalent total lethal time *M_ref_* (min) for a specific temperature–time history of *T*(*t*) can be calculated by the cumulative thermal inactivation rate of this thermal treatment using the following integral equation [19,27]:

(4) $$M_{\mathrm{ref}}=\int_{0}^{t}{\;10\;}^{\frac{T\left(t\right)-T_{\mathrm{ref}}}{z}}\;\mathrm{dt}$$

where *z* is in the thermal inactivation time curve, the temperature difference (°C) required for reducing 1 log population. The *z*-value can be calculated based on the following equation [28]:

(5) $$z=\frac{T_{2}-T_{1}}{{\log\;D}_{1}-{\;\log\;D}_{2}}$$

where *D*_1_ and *D*_2_ are the decimal reduction times (min) of target molds under temperatures (°C) of *T*_1_ and *T*_2_. The *z*-value could be defined as the ratio of the difference in the log *D*-values to the difference in the exposure temperatures.

### 2.6. RF Pasteurization Validation

#### 2.6.1. Inoculated Almond Samples

The *a_w_* of almond kernel samples was adjusted to 0.657, 0.854 and 0.923, respectively. Each almond kernel sample with different *a_w_* was first exposed to ultraviolet lights for at least 1 h and turned over every 30 min [13]. Then, about 5 g (5 ± 0.2 g) sterilized almond kernels with different *a_w_* were put in sterile polyethylene bag (5 × 7 cm^2^), and 20 μL target mold suspension was inoculated into the sterile bag. All the bags were rubbed at least 3 min by hand to make the suspension evenly attached to the almond kernels’ surface [29,30]. Inoculated almond kernels were left for 12 h at 23 ± 2 °C inside a biosafety hood to achieve a sufficient moisture equilibration and then wrapped in sterile filter paper, and tied with a rubber band [31]. The final populations of target mold on different *a_w_* almond kernel samples were achieved at 10^7^ CFU/g.

#### 2.6.2. Selection of Electrode Gap

**Figure 2:**
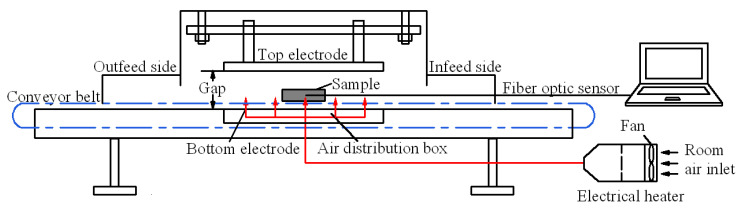
Schematic view of the pilot-scale 6 kW, 27.12 MHz RF system (Adapted from Wang et al. [33]).

**Figure 3:**
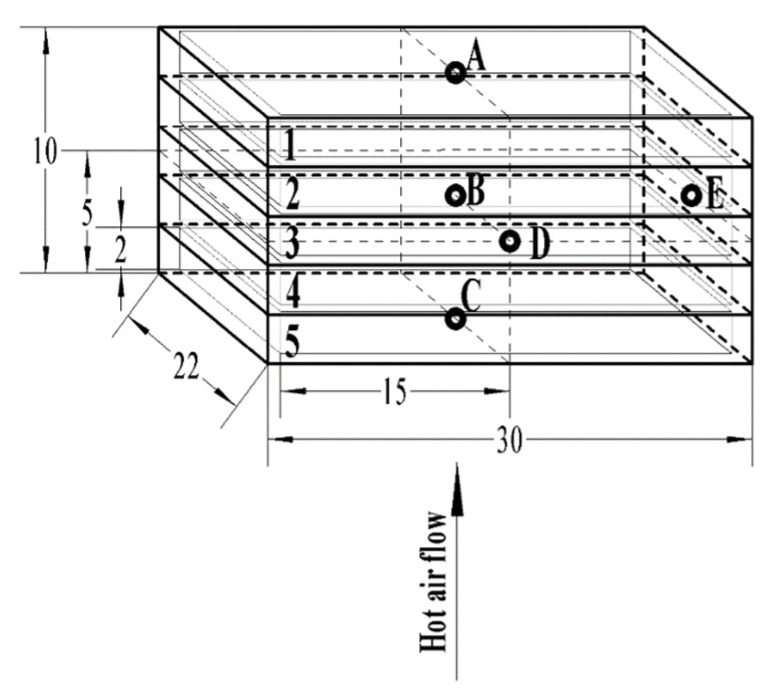
Five-layer (1–5) container for sample temperature measurements with five positions (A–E) and pre-drilled holes (all dimensions are in cm) (Adapted from Li et al. [32]).

Each 1.5 kg of almond kernel with *a_w_* of 0.657, 0.854 and 0.923 were placed homogeneously into the uncovered five-layer container, respectively (300 g almond kernel for each layer). Detailed information on a five-layer container can be found in Li et al. [32]. Then, the five-layer container containing 1.5 kg pretreated almond kernels was placed vertically above the bottom electrode of the RF system (Figure 2) to obtain the general relationship between the electrode gap and current (I, A). The RF system’s detailed information can be found in Wang et al. [33]. Based on the anode current (I, A) shown on the RF system screen, the output RF power (P, kW) was calculated according to the equation of P=5× I −1.5 recommended by the manufacturer, and the heating rates of the almond kernels were estimated [34,35]. The heating rate of each location and the location of the coldest spot were determined by inserting probes into the almond kernels through pre-drilled holes in five representative locations (A–E) (Figure 3) using a fiber optic temperature sensor system (HQ-FTS-D120, Heqi Technologies Inc., Xian, China). According to the similar heating rate around 6.7 °C/min during RF heating, the electrode gaps of 10.5 cm, 12.5 cm and 13.0 cm were finally selected for the almond kernels with *a_w_* of 0.657, 0.854 and 0.923, respectively.

#### 2.6.3. RF Pasteurization Verification

Based on the target mold’s thermal inactivation kinetics in almond kernels, the hot air-assisted RF system was used for pasteurization verification. The temperatures of 71, 68, and 65 °C were selected, respectively, as the target holding temperatures of almond kernels with *a_w_* of 0.657, 0.854 and 0.923 for pasteurization validation. Every four packs of filter-paper-wrapped inoculated almond kernels with three different *a_w_* were placed at cold point (point B of Layer 3, Figure 3) in the five-layer container, respectively [32]. Then, the five-layer container was placed above the bottom electrode of the hot air-assisted RF system and heated with the appropriate electrode gap until the temperature of the cold spot reached the target value. The RF system was then switched off and the almond kernels were kept at the target temperature only by hot air. To ensure the heating uniformity during RF pasteurization, the position order of the five-layer container was rearranged from L1, L2, L3, L4, and L5 to the order of L5, L4, L3, L2, and L1 according to Li et al. [32]. The hot air holding temperatures of almond kernels with *a_w_* of 0.657, 0.854 and 0.923 were set to 74, 71, and 68 °C, respectively, slightly above the target temperature based on the thermal loss during heating [13].

According to the different *D*-values of target mold under different *a_w_* and temperature levels, the packs were taken out at different time intervals, sealed with polyethylene bags, and then immersed in cold water below 4 °C for at least 3 min for fully cooling. The pack wrapped in inoculated but no-treated almond kernels was conducted for plate counting to detect the total numbers of molds before thermal treatment. Specifically, the almond kernels were put into normal saline (10 mL) and shaken for 3 min sufficiently. The target mold suspensions were then diluted by gradient dilution and appropriate dilutions were selected to count the population of the sample colonies. The validation test was repeated three times for each *a_w_*.

### 2.7. Statistical Analysis

Each trial was performed for three biologically separate replicates. Analysis of variance (ANOVA) and Tukey’s test (*p ≤* 0.05) were used for evaluating the statistical significance of differences. SPSS statistics 21.0 software (IBM, Armonk, NY, USA) was used for performing model fitting and parameter estimations.

## 3. Results and Discussion

### 3.1. Spoilage Molds Isolated from Almond Kernels

Colonies appeared after three to four days of inoculation on both CYA and PDA media. A cyan mold and a black mold were separated and purified in CYA media and PDA media, respectively. The cyan mold was identified as *Penicillium* and the black mold was identified as *Aspergillus* after WGS by the identification mechanism (Sangon Biotech Co., Ltd., Shanghai, China).

### 3.2. Selection of the More Thermal-Resistant Mold

Table 1 showed the population reductions of *Penicillium* and *Aspergillus* inoculated into almond kernel flour with an *a_w_* of 0.854 under three combinations of heating temperature and time. The population reductions of *Penicillium* were higher than those of *Aspergillus* (*p ≤* 0.05), suggesting that the selected *Aspergillus* had higher heat resistance than the selected *Penicillium* in almond kernels. Therefore, *Aspergillus* was selected as the target mold to explore the influence of different *a_w_* levels and temperatures on the thermal inactivation kinetics.

### 3.3. Primary Model

Table 2 presented the *D*-, *δ*- and *p*-values of the two models for the target *Aspergillus* in almond kernel flour at three *a_w_* and temperature levels. The Weibull model’s coefficients of determination (R^2^ = 0.988–0.998) were higher than those (0.935–0.992) of the first-order kinetic model, and the Weibull model’s root mean square errors (RMSE = 0.056–0.181) were lower than those (0.153–0.503) of the first-order kinetic model. The Weibull model was more appropriate for describing the survival curves of the target *Aspergillus* in almond kernels when compared with the first-order kinetic. All the Weibull model’s *p*-values were less than 1, indicating a tailing behavior of the curves. This might be due to the fact that with the temperature increasing, the surviving mold had stronger heat resistance, or was more adaptable with treatment time [36]. Dong [37] and Zhang et al. [8] also reported similar results in *Clostridium sporogenes* and *Aspergillus flavus*.

**Figure 4:**
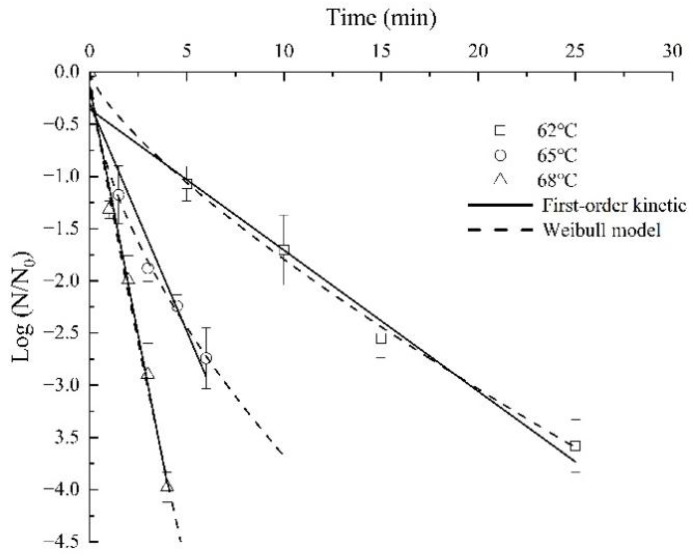
The target *Aspergillus* inactivation from the first-order kinetic and the Weibull models affected by temperature under *a_w_* of 0.854.

At a specific *a_w_* value, the *D*-values were dependent on the sample temperature, that was, when the temperature was higher, the shorter time needed for achieving the target *Aspergillus*’ inactivation rate. As an example, at *a_w_* of 0.854, when the temperature was 62 °C, the *D*-value was 7.09 min, but the *D*-values dropped to 2.29 min and 1.05 min at 65 °C and 68 °C, respectively. The Weibull model’s *δ*-values also decreased with the temperature increase, which indicated that as the temperature increased, the target *Aspergillus*’ thermal inactivation rate increased. For example, at 62 °C, the *δ*-value was 4.64 min when *a_w_* was 0.854 but sharply declined to 1.10 min at 65 °C and 0.85 min at 68 °C. The tendency was in agreement with *Acidovorax citrulli* on watermelon seeds [26], *E. coli ATCC 25922* in mashed potato [38], and *Salmonella enterica* in goat’s milk caramel [39]. The target *Aspergillus* inactivation from the first-order kinetic and the Weibull models affected by temperature under *a_w_* of 0.854 were shown in Figure 4. The slope of the curves increased with the increase in temperatures, and also showed that the lower the temperature, the more obvious the tailing effect, which is corresponding to Table 2.

**Figure 5:**
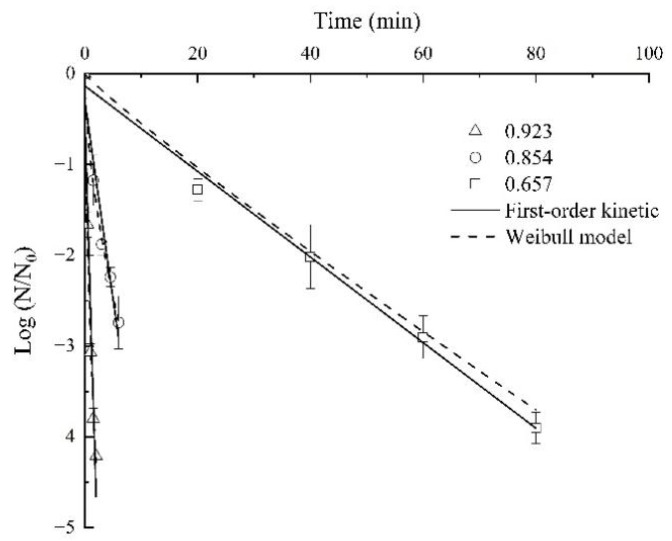
Survival curves of *Aspergillus* at 65 °C with *a_w_* of 0.657, 0.854 and 0.923, by fitting with first-order kinetic and Weibull models.

The *D*-values and the *δ*-values both decreased with the increase in *a_w_* at the same temperature. For example, when the temperature was 65 °C and *a_w_* was 0.657, the *D*-values were 21.82 min and the *δ*-values were 19.28 min. However, when *a_w_* increased to 0.854 and 0.923, the *D*-values were reduced to 2.29 min and 0.48 min, and *δ*-values also decreased to 1.10 min and 0.17 min, respectively. Zhang et al. [40] also displayed that the thermal treatment time could be effectively shortened and the ideal microbial inactivation level could be achieved in a short time with the increase in *a_w_* levels. For example, the time required to reduce the populations of the target *Aspergillus* in almond kernel flour by 4 log at 65 °C calculated from the Weibull model, 77.12 min, 4.40 min and 0.68 min were needed when the *a_w_* of almond kernels was 0.657, 0.854 and 0.923, respectively. Figure 5 showed the survival curves of *Aspergillus* at 65 °C with *a_w_* of 0.657, 0.854 and 0.923, by fitting with first-order kinetic and Weibull models. The survival curve of *Aspergillus* with *a_w_* of 0.923 was relatively straight, and the survival curves of *Aspergillus* with *a_w_* of 0.854 and 0.657 were slightly upward.

According to the data in Table 2, the *p*-value of the shape parameter appeared to be independent of *a_w_* and temperature, which is consistent with the previous results [18,41]. The re-estimated *δ’*-values at the mean of survival curves with the *p*-value fixed to 0.70 are shown in Table 3. The re-estimated *δ’*-values ranged from 0.23 min to 13.40 min, which were influenced by the test temperature and sample *a_w_* as explained by Possas et al. [42].

### 3.4. Secondary Model

Table 4 presented the *D_ref_*, *z_aw_*, and *z_T_* values of Mafart’s modified Bigelow model calculated at 65 °C using the data from first-order kinetic and the Weibull model for the thermal inactivation of *Aspergillus* inoculated into the almond kernels. The Mafart’s modified Bigelow model conforms to the first-order kinetic model (R^2^ ≥ 0.932 with RMSE ≤ 0.150), or the Weibull model for related *p*-value (R^2^ ≥ 0.853 with RMSE ≤ 0.256) and for single *p*-value (R^2^ ≥ 0.907 with RMSE ≤ 0.182). Combined with the estimated parameters from Table 4 and Equation (3), the thermal inactivation results of the target *Aspergillus* under any given treatment temperature and *a_w_* conditions within the experimental limits can be predicted.

### 3.5. Electric Current under Different Electrode Gaps

**Figure 6:**
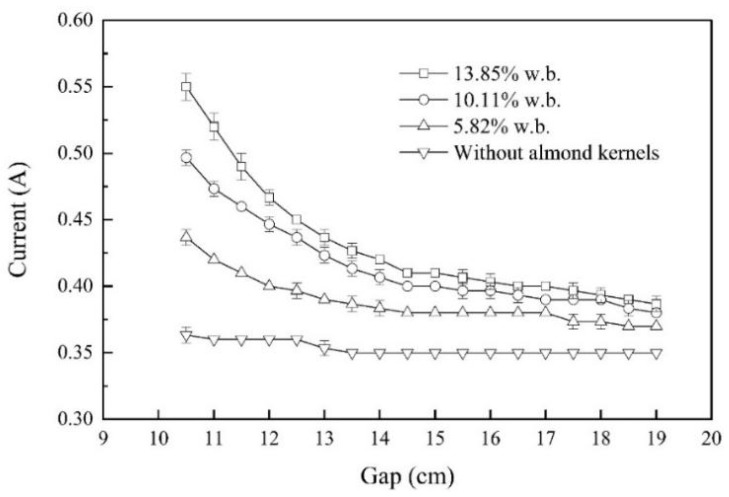
The relationship between electric current and electrode gap for almond kernels with three different *a_w_* levels without conveyor belt movement and hot air-assisted heating.

The relationship between electric current and electrode gap without conveyor belt movement and hot air-assisted heating was shown in Figure 6. In the five-layer container, the electric current gradually decreased as the electrode gap increased from 10.5 cm to 19.0 cm, which is similar to the previous research results [43,44]. Because of the same output power calculated by the same electric currents, the 10.5 cm, 12.5 cm and 13.0 cm electrode gaps of almond kernels with *a_w_* of 0.657, 0.854, and 0.923 were selected, respectively, to achieve similar heating rates in RF heating process. The heating rates measured by the fiber optic temperature sensor system under the corresponding electrode gap were 6.54 ± 0.12, 6.84 ± 0.16 and 6.65 ± 0.17 °C/min, respectively.

### 3.6. Cumulative Lethal Effect of Aspergillus

**Figure 7:**
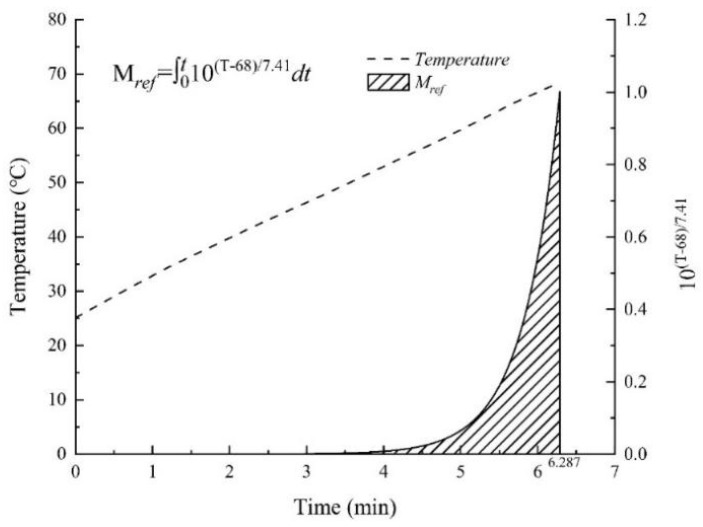
Average temperature–time history of five locations (A–E) in Figure 3 of RF heating from 25 to 68 °C, and the equivalent lethal time *M_ref_* for this heating up curve of *Aspergillus* inoculated in almond kernels with *a_w_* of 0.854 at 68 °C.

The target molds’ thermal inactivation kinetics is built under isothermal conditions. However, in practical production and application, most thermal treatment processes were of non-isothermal characteristics. The average temperature–time history of 1.5 kg almond kernels with 0.854 *a_w_* (10.11% w.b. MC) in the five positions (A–E) of the five-layer container with a 12.5 cm electrode gap was shown in Figure 7. To design the RF treatment processes for almond kernels’ pasteurization according to the thermal inactivation kinetics of the target *Aspergillus*, the heating up processes should be transformed into isothermal processes based on the cumulative lethal effect model depicted in Equation (4). The target *Aspergillus*’ *z*-value was estimated to be 7.41 °C when *a_w_* was 0.854 according to the data in Table 2. At the reference temperature of 68 °C, the equivalent lethal time *M_ref_* of the RF heating up process curve (from 25 °C to 68 °C) was the area of the shaded part (0.471 min) in Figure 7. When *a_w_* values were 0.657 and 0.923, the cumulative thermal lethal time during heating up were 0.392 and 0.367 min at the reference temperature of 71 °C and 65 °C, respectively. In a certain thermal process, when the temperature increases, the cumulative thermal curve becomes steeper and steeper, which was the same as the result obtained by Zhang et al. [8]. Theoretically, the *D*_68°C_-value of *Aspergillus* in almond kernels with an *a_w_* value of 0.854 was 1.05 min (shown in Table 1). To obtain 4 log reductions of *Aspergillus*, almond kernels need to be heated continuously at 68 °C for 4.20 min. As shown in Figure 7, there would be an additional 3.73 min holding time required in this thermal process to obtain the 4 log reductions of the target *Aspergillus*.

### 3.7. RF Treatment Verification

**Figure 8:**
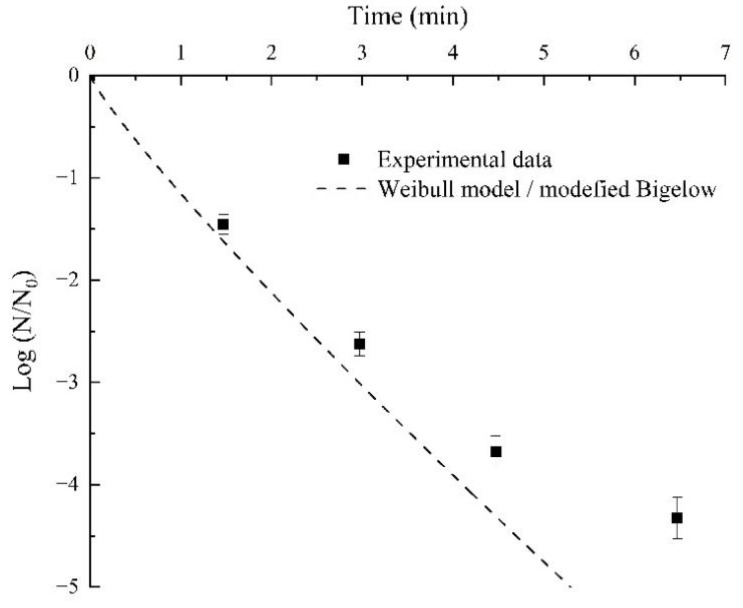
Experimental data and predicted survival curves for the target *Aspergillus* inoculated into almond kernel flour with 0.854 *a_w_* at 68 °C by combining the Weibull model with Mafart’s modified Bigelow equation.

Figure 8 showed the experimental data for verifying almond kernels’ RF pasteurization levels and the predicted survival curve for the target *Aspergillus* inoculated into almond kernel flour with *a_w_* of 0.854 at 68 °C by combining the Weibull model with the Mafart’s modified Bigelow equation. The time shown on the abscissa in Figure 8 was the sum of the cumulative thermal lethal time calculated by the heating up process and the time of the isothermal thermal process. The results showed that the RF pasteurization verification time was slightly longer than the time predicted by the combined cumulative thermal lethal time and the isothermal heating time.

The longer time required in validated RF pasteurization may be due to the difference in the particle size. When obtaining thermal inactivation kinetics of *Aspergillus*, the *Aspergillus* suspension was inoculated in the almond kernel flour, while the *Aspergillus* suspension was inoculated on the whole almond kernels when validated in the RF system. These results were the same as those in previous research. For example, Fine et al. [45] found that the *Saccharomyces cerevisiae* in larger size wheat flour exhibited higher heat resistance. Zhang et al. [18] also observed that the *E. coli ATCC 25922* inoculated in pepper powder behaved more thermal resistant with the increase in the particle size of pepper powder. In addition, the *Aspergillus* in the almond kernels may be more thermal resistant than in the almond kernel flour because it takes time for central heat to diffuse to the surface of almond kernels.

For validating RF pasteurization, as the heating time was prolonged, the MC of almond kernels gradually declined, which enhanced the heat resistance of *Aspergillus*. This phenomenon was consistent with that in a previous study. For example, Li et al. [32] found that the heat resistance of *E. coli ATCC 25922* inoculated in almond kernels increased with the increase in RF heating time. Chen et al. [46] also found that the rapid evaporation of water on the hard-shell surface of hazelnuts with a shell led to the unsatisfactory inactivation effect of *Salmonella*.

## 4. Conclusions

*Penicillium* and *Aspergillus* were identified from moldy almond kernels by WGS. The selected *Aspergillus* had higher heat resistance than the *Penicillium* in almond kernels. The thermal inactivation kinetics of *Aspergillus* in almond kernel flour affected by temperature and *a_w_* was studied and then fitted by using the first-order kinetics and Weibull models. The Weibull model was more appropriate when characterizing the survival curves of the target *Aspergillus* in almond kernels due to the higher coefficients of determination and lower root mean square errors. The *D_ref_*, *z_aw_,* and *z_T_* values from Mafart’s modified Bigelow model were calculated and used for predicting the thermal inactivation of *Aspergillus* under any given treatment temperature and *a_w_* conditions. The predicted thermal inactivation kinetic models were verified by RF heating in combination with the cumulative thermal lethal model. The results showed that RF pasteurization verification time was slightly longer than the time predicted by the combined cumulative thermal lethal time and the isothermal heating time due to the different particle sizes and other possible factors. Future studies may focus on the effect of real-time moisture content change on microbial heat resistance in almond kernels under RF treatment.

## Figures and Tables

**Table 1:** Population reductions (mean ± SD, log CFU g^−1^) of *Penicillium* and *Aspergillus* inoculated in almond kernel flour with an *a_w_* of 0.854 under the three treatment conditions.

Types of Molds	Temperature (°C) + Holding Time (min)		
	62 °C + 7 min	65 °C + 3 min	68 °C + 1 min
*Penicillium*	2.07 ± 0.07 ^a^^,^*	3.52 ± 0.21 ^a^	1.55 ± 0.13 ^a^
*Aspergillus*	1.42 ± 0.12 ^b^	2.21 ± 0.11 ^b^	1.10 ± 0.17 ^b^

* Different letters in the same column indicate that there were significant differences in the values of the population reductions with *p* < 0.05 between the two molds.

**Table 2:** *D*-, *δ*- and *p*-values of the two models for the target *Aspergillus* in almond kernel flour at three *a_w_* and temperature levels using test cells.

Moisture Content (% w.b.)	*a_w_*	Temperature (°C)	First-Order Model			Weibull Model			
			*D* (min)	R^2^	RMSE	*δ* (CI 95%) ^a^	*p* (CI 95%)	R^2^	RMSE
5.82	0.657	65	21.82	0.992	0.153	19.28 (12.21–26.34)	0.92 (0.65–1.20)	0.992	0.150
		68	7.28	0.966	0.356	3.73 (2.21–5.25)	0.70 (0.54–0.85)	0.995	0.135
		71	2.10	0.968	0.315	1.15 (0.54–1.75)	0.70 (0.48–0.92)	0.993	0.151
10.11	0.854	62	7.09	0.980	0.222	4.64 (3.59–5.69)	0.76 (0.64–0.88)	0.997	0.082
		65	2.29	0.946	0.285	1.10 (0.82–1.37)	0.59 (0.49–0.69)	0.998	0.056
		68	1.05	0.991	0.167	0.85 (0.53–1.18)	0.88 (0.63–1.13)	0.993	0.149
13.85	0.923	59	5.43	0.979	0.249	3.27 (2.00–4.55)	0.73 (0.55–0.92)	0.995	0.118
		62	2.45	0.953	0.331	1.14 (0.34–1.94)	0.63 (0.36–0.89)	0.988	0.168
		65	0.48	0.935	0.503	0.17 (0.03–0.30)	0.59 (0.38–0.80)	0.992	0.181

^a^ CI 95%: Confidence Interval.

**Table 3:** The re-estimated *δ’*-values at the mean of survival curves with the *p*-value fixed to 0.70.

Moisture Content (% w.b.)	*a_w_*	Temperature (°C)	*δ’* (CI 95%) ^a^	R^2^	RMSE
5.82	0.657	65	13.40 (10.95–15.85)	0.973	0.238
		68	3.76 (3.47–4.06)	0.995	0.117
		71	1.14 (1.04–1.25)	0.993	0.131
10.11	0.854	62	4.12 (3.79–4.45)	0.995	0.098
		65	1.37 (1.23–1.50)	0.991	0.103
		68	0.62 (0.53–0.72)	0.980	0.213
13.85	0.923	59	3.04 (2.81–3.28)	0.995	0.108
		62	1.35 (1.18–1.52)	0.985	0.162
		65	0.23 (0.20–0.26)	0.984	0.216

^a^ CI 95%: Confidence Interval.

**Table 4:** Calculated *D_ref_*, *z_aw_*, and *z_T_* values of Mafart’s modified Bigelow model at 65 °C for the thermal inactivation of *Aspergillus* inoculated into the almond kernels.

Parameter	First-Order Kinetic Model	Weibull Model	
		*δ*	*δ’*
*D_ref_* or *δ_ref_* (min)	0.326	0.140	0.173
*Z_T_* (°C)	6.660	6.130	6.493
*z_aw_*	0.189	0.169	0.185
R^2^	0.932	0.853	0.907
RMSE	0.150	0.256	0.182

## Data Availability

The data presented in this study are available in this article.
